# A novel kit for early diagnosis of Alzheimer’s disease using a fluorescent nanoparticle imaging

**DOI:** 10.1038/s41598-019-49711-y

**Published:** 2019-09-12

**Authors:** Jun Sung Park, Sang Tae Kim, Sang Yun Kim, Min Gi Jo, Myeong Jun Choi, Myeong Ok Kim

**Affiliations:** 1 Division of Life Science and Applied Life Science (BK21 plus), College of Natural Sciences, Gyeongsang National University (GNU), Jinju, 52802 Republic of Korea; 2 Department of Neurology, Seoul National University Bundang Hospital, Seongnam-si, Gyeonggi-do 13605 Republic of Korea; 3 Research and Development Center, Phytos Inc, Anyang mega valley 609, 268 Anyang, Gyeonggi-do Republic of Korea

**Keywords:** Antisense elements, Antisense elements, Antisense elements, Alzheimer's disease, Alzheimer's disease

## Abstract

Alzheimer’s disease (AD) is a progressive neurodegenerative disease and chronic illness with long preclinical phases and a long clinical duration. Until recently, a lack of potential therapeutic agents against AD was the primary focus of research, which resulted in less effort directed towards developing useful diagnostic approaches. In this study, we developed a WO2002/088706 kit that is composed of fluorescent nanoparticles for the early detection of AD. We provided a fluorescent nanoparticle for detecting markers and a kit for the early diagnosis of AD. The kit consists of a probe molecule comprising an oligonucleotide capable of detecting one or more AD-specific microRNAs (miRNAs) and biomarkers related to AD. Through screening, we selected miR-106b, miR-146b, miR-181a, miR-200a, miR-34a, miR-124b, miR-153, miR-155, Aβ_1-42_ monomer (mAβ), Aβ_1–42_ oligomer (oAβ), UCHL1, NLRP3, Tau, STAT3, SORL1, Clusterin, APOE3, APOE4, Nogo-A, IL-13, and Visfatin to serve as AD- and inflammation-related markers. For detection of kit-binding properties, we checked the expression levels of amyloid beta (Aβ), tau protein, and inflammatory mediators in APP/PS/ApoE knockdown (KD) mice and a control group using co-localisation analysis conducted with a confocal microscope. Using a similar approach, we checked the expression levels of miRNAs in HT22 cells. Finally, we used the plasma from AD patients to confirm that our fluorescent nanoparticles and the WO2002/088706 kit will provide a possible early diagnosis to serve as an AD detector that can be further improved for future studies on targeting AD.

## Introduction

Alzheimer’s disease (AD) is a neurodegenerative disease with the highest incidence and is one of the most common central neurological disorders^1,2^. According to the World Health Organization (WHO), in the middle of 2015, more than 36 million people worldwide were suffering from AD. High expression of inflammatory mediators has been demonstrated in the area of Aβ peptide deposits and neurofibrillary tangles, especially in patients with AD^3,4^. Various other studies have demonstrated a significant increase in proinflammatory cytokines and chemokines in the brains of AD patients^5,6^. Until now, there has been no precise diagnostic approach for treating AD^7,8^.

There are two dominant hallmarks associated in AD^9^. One is the accumulation of insoluble amyloid beta (Aβ), which forms plaques in extracellular spaces and the walls of blood vessels^10,11^. The other is the aggregation of hyperphosphorylated tau that forms neurofibrillary tangles in neurons^10–12^. The amyloid precursor protein (APP) cleaved by α- and γ-secretases produces no aggregating fragments under normal conditions^13^. However, the APP molecules cleaved by β- and γ-secretases produce Aβ, which can form Aβ plaques^13^. Aβ plaque formation is a major pathological event in the brains of AD patients and results in cognitive and memory dysfunction. Aβ acts as a neurotoxin by initiating a group of biochemical cascades that lead to synaptic toxicity and neurodegeneration^14–16^. The increased aggregation of the phosphorylated tau protein decreases microtubule binding, leading to axonal transport dysfunction and neuronal loss. Nevertheless, the molecular mechanisms underlying Aβ accumulation, tau phosphorylation, synaptic loss and neurodegeneration remain unknown^7,8^.

Various therapeutic agents against AD have been established in the past few years, including therapies that promote inhibition of secretase activity, Aβ clearance from the brain, and protection of neurons to correct neuronal function; nanoparticulate systems have also been developed^17–20^. Recently, various nanoparticles have been used for drug delivery systems that transfer therapeutics to a specific region and ameliorate various neurodegenerative disorders to reduce neurotoxic effects in the brain^21,22^.

Nanotechnological advances have recently been explored in new studies to overcome the effects of neurological disorders^23,24^. For the treatment of severe diseases, nanotechnologies such as nanoparticulate systems have potential and may provide the best approach^25,26^. However, there have been symptomatic studies of therapeutics for AD, and many of them were considered failures due to a lack of experimental evidence demonstrating clinical benefits. The most likely explanation for those failures is that the drugs were administered too late for the treatment of AD and neuropathological disorders^27^. The therapy will be more effective if delivery is during the preclinical stage of AD before brain damage occurs, and detection of earlier biomarkers is needed to know when to deliver the drugs to the brain^28^. Methods for determining the preclinical stage for better outcomes of AD treatment is still being explored, and further studies are needed^29,30^. Therefore, to facilitate monitoring for AD, there is a prodigious demand for the invention of methods for early-stage diagnosis of AD.

Currently, efforts are being made to develop diagnostic indices of AD^31^. These efforts include the development of the AD Diagnostic Kit WO2002/088706, which uses glutamine synthetase as an index, and WO2010/144634, which diagnoses AD by confirming the degree of DNA methylation as an epigenetic marker.

A previous study demonstrated the expression of miRNAs in AD pathology that may contribute to AD pathogenesis^32^. In this study, we provided a fluorescent nanoparticle for detecting antigens and a kit for the early diagnosis of AD that consists of a probe molecule comprising an oligonucleotide that can detect one or more AD-specific miRNAs and antigens. If you know the specific factor for AD, it can be measured using the kit from this study. Therefore, the potential of this modality is expected to be infinite.

Traditional methods, FISH (fluorescence *in situ* hybridisation) and ELISA (enzyme-linked immunosorbent assay), require complex production techniques, high cost of experimentation and time consuming. However, this kit provides faster diagnostic results with less complex production techniques and lower costs. In addition, the greatest advantage of the kit is that it is highly accessible to patients because it is diagnosed through blood^33,34^. In the presence of target molecules in blood or tissues in Tris-HCl + NaCl (pH 7.2) buffer, the loop is opened due to strong affinity with the target, resulting in fluorescence due to the distance between the quantum dot and the quencher. Hence, unlike traditional methods, this method of diagnosing Alzheimer’s disease is expected to be convenient, inexpensive and fast.

## Results

### Physical and binding properties of the diagnostic kit

Studies have been conducted to develop diagnostic indices for the early detection of AD^35^. Our fluorescent nanoparticle complex for detecting miRNA and antigens for the early detection of AD has the following properties and structure.

**Figure 1:**
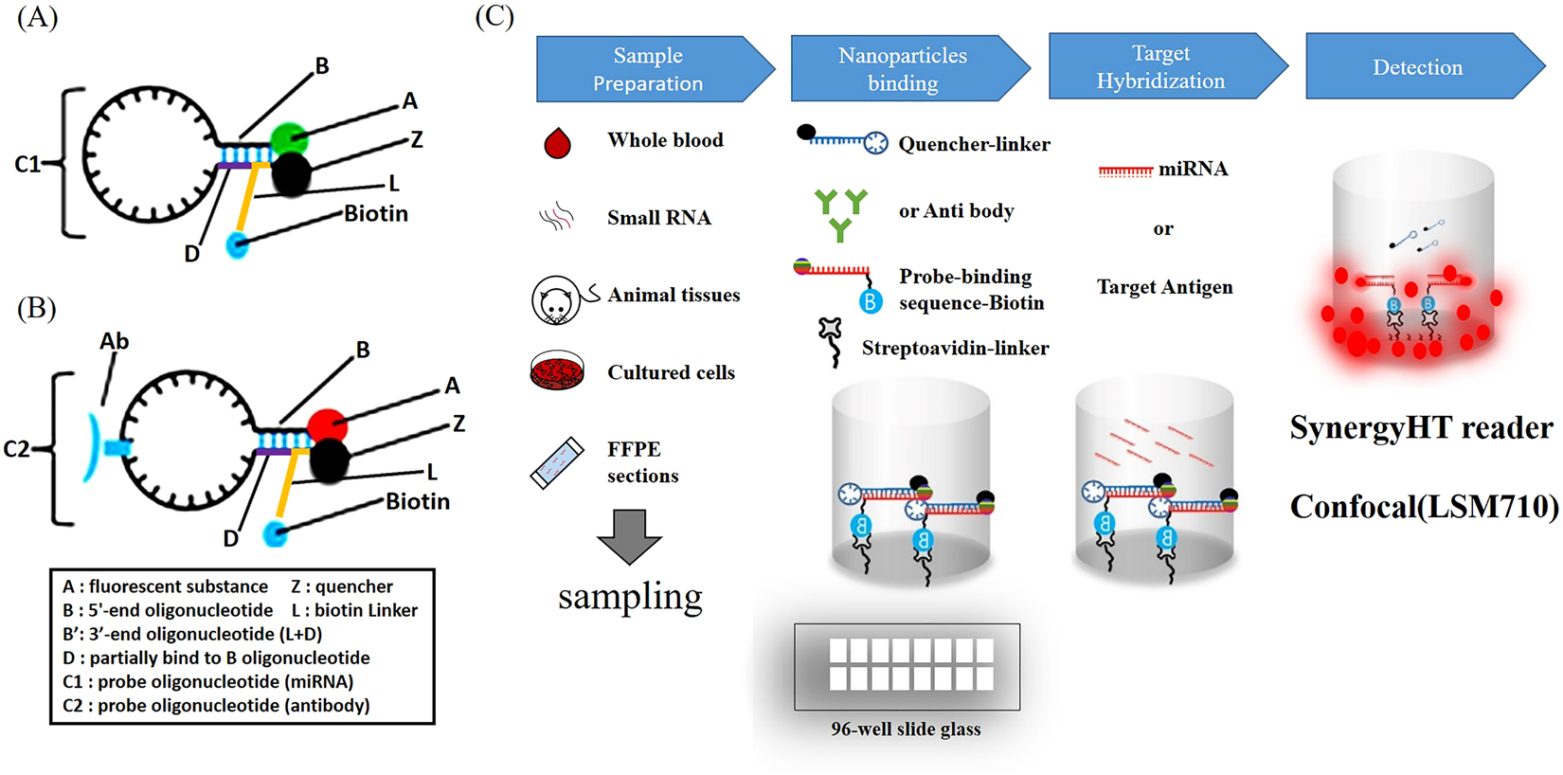
The development of the WO2002/088706 fluorescent nanoparticle kit and application of the nanoparticles. Figure illustrating the structure of a probe complex according to the design for this study. Figure showing the state in which fluorescent nanoparticles are bound to regions that can be dissociated when binding to specific target molecules in samples such as nerve cells, tissues or plasma. The figure also illustrates the overall process and outline of the present study. (**A**) Nanoparticle complexes for miRNA detection. (**B**) Nanoparticle complexes for antigen detection. (**C**) Schematic view illustrating a reaction process in a plastic container of a fluorescence sensor that can detect an antigen and a specific miRNA in the early stages of AD using the probe complex designed for this study. First, the samples of tissues, plasma, nerve cells or small RNA were prepared as shown in (**C**). The complexes in (**A**) or (**B**) were added to streptavidin-coated glass, and the biotin and streptavidin contained in the complexes bound to each other. By adding the miRNA or antigen, target hybridisation occurred, and the results were detected using a Synergy HT reader and a confocal microscope.

First, the molecules of structures I and II form a fluorescent nanoparticle complex for AD-specific miRNA detection and have a structure of A-B-C1-B’-Z (Fig. 1A).

Alternatively, the kit can include two probe molecules each with structure I or II.

#### A-B-C1-D (I), Z-L (II)

In this structure, A is a fluorescent substance, and B is a 5′-end oligonucleotide of 3 to 10 nt. B’ is a complementary oligonucleotide binding with B. C1 is a probe oligonucleotide that can bind to AD-specific microRNAs in a complementary manner while forming a loop. D is a nucleotide that can partially bind to B in a complementary manner and serves as a switch to thermodynamically dissociate Z from A when the target miRNA or antigen is bound to the probe oligonucleotide. L is capable of partial complementary binding with B. L is also a linker region that forms a stem together with D and can bind to biotin. B’ includes partial L and D. Z is a quencher capable of cancelling the fluorescence of A. If AD-specific microRNA molecules are absent, B, C, D, and L form a stem-loop structure, and the fluorescence of A is quenched by Z.

Second, molecules with structures III and II form a fluorescent nanoparticle complex for AD-specific antigen detection and have the structure of A-B-Y-C2-B’-Z (Fig. 1B).

#### A-B-Y-C2-D (III), Z-L (II)

In this structure, A is a fluorescent substance, and B is a 5′-end oligonucleotide of 3 to 10 nt. B’ is a complementary oligonucleotide binding with B. C2 is an oligonucleotide that has an antibody that binds in a complementary manner to an AD-specific antigen while forming a loop. D is a nucleotide that can partially bind to B in a complementary manner and serves as a switch to thermodynamically dissociate Z from A when the target miRNA or antigen is bound to the probe oligonucleotide. L is capable of partial complementary binding with B. L is also a linker region that forms a stem together with D and can bind to biotin. B’ includes partial L and D. Z is a quencher capable of cancelling the fluorescence of A. If AD-specific microRNA molecules are absent, B, C, D, and L form a stem-loop structure, and the fluorescence of A is quenched by Z.

In the above diagnostic kit, the AD-specific microRNAs are miR-106b, miR-146b, miR-181a, miR-200a, miR-34a, miR-124b, miR-153, and miR-155, and the AD-specific antigens are Aβ_1–42_ monomer, Aβ_1–42_ oligomer, UCHL1, NLRP3, Tau, STAT3, SORL1, Clusterin, APOE3, APOE4, Nogo-A, NF-kB, IL-13, and Visfatin.

Based on this basic structure, we can make a molecular beacon-based sensor through a series of processes (Fig. 1C). The most important features of this processing include the following principles. Since the quencher is usually conjugated with the 5′-end oligonucleotide or the 3′-end oligonucleotide by the linker region at a position near the fluorescent substance, the fluorescent substance is quenched by the quencher. However, when the AD-specific miRNA or antigen in the sample is bound to the probe molecule or the nanoparticle complex, the stem-loop structure is released, and the fluorescent substance and the quencher are separated from each other, which allows fluorescence to be generated by the fluorescent substance.

In other words, a molecular fluorescence detection system can be provided in which a signal-off state occurs when target molecules are not present and a signal-on state occurs when target molecules encounter the nanoparticle complex.

Therefore, when this molecular image sensor is used, the presence or absence of a specific target molecule in a cell or tissue can be confirmed using a simple *in vitro* method. In addition, the presence or absence of the target molecule can be confirmed by turning the fluorescent signal on and off after transfection into the cell or tissue targeted by the nucleic acid sensor.

In addition, the kit also includes a reaction vessel coated with streptavidin, and the reaction vessel may be a 24-well, 48-well, 96-well, 192-well or 384-well microplate. Quantum dots can be formed with the diagnostic kit, and fluorescence can be selected from a group consisting of poly-lysine fluorescein isothiocyanate (FITC), tetramethyl rhodamine-B-isothiocyanate (TRITC), and rhodamine-6-carboxy-X-rhodamine (ROX) as rhodamine derivatives, as well as 6-carboxyrhodamine (R6G), lissamine rhodamine B, sulfonyl chloride, rhodamine B, rhodamine 123, rhodamine X isothiocyanate, sulforhodamine B, sulforhodamine 101, sulfonyl chloride rhodamine (Texas Red) of sulforhodamine 101, N,N,N′,N′-tetramethyl-6-carboxyrhodamine (TAMRA), tetramethyl rhodamine, tetramethyl rhodamine isothiocyanate (TRITC), riboflavin, rosolic acid, erbium chelate derivatives, Alexa derivatives, Alexa-350, Alexa-488, Alexa-547, Alexa-647, Cy2, Cy3, Cy5 and quantum dots (QD).

The structure includes a polymer coating layer around the central part, and the central part encloses the centre body. For that technique, the kind of quantum dots is not particularly limited, and any quantum dot can be used without limitation as long as it is biocompatible with live imaging. For the current approach, quantum dots with a particle diameter of 5 nm or less can be used. The constituents of the centre body of the quantum dots are mainly heavy metals, such as CdSe (cadmium selenide), CdTe (cadmium telluride) or CdS (cadmium sulfide).

### Screening of AD-related markers

**Figure 2:**
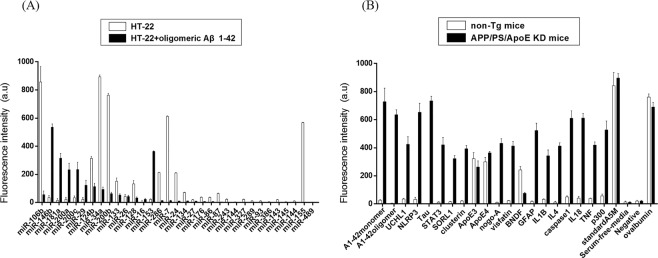
Screening of AD antigens and miRNAs via image fluorescence sensors *ex vivo* and *in vitro*. (**A**) Graph showing changes in the expression pattern of miRNAs in HT22 cells treated with or without Aβ_1–42_ oligomer. The miRNA levels were evaluated by microarray analysis using antisense oligonucleotide chips specific for miRNAs. (**B**) Graph showing changes in the expression patterns of various inflammation-related antigens and AD-related antigens in the plasma of non-Tg and AD-induced model mice (APP/PS/ApoE knockout mice).

We have identified the inflammatory response factors that are produced by the accumulation of Aβ during AD induction to find candidates that can be used as diagnostic markers for AD. First, the level of inflammation-specific miRNAs in HT22 cells, which are a hippocampal cell line, treated with or without Aβ_1–42_ oligomers was analysed using microarrays containing miRNA-specific antisense oligonucleotides (Fig. 2A and Table 1). Second, to confirm the serologic markers in an animal model of AD, immunochemical analysis was performed on the plasma of the normal group of non-transgenic mice (6 animals) and the APP/PS/ApoE knockdown mice for the AD animal model (6 animals) as well as separate biomarkers related to AD (Fig. 2B and Table 2).

**Table 1:** Comparison of miRNA expression levels in control and Alzheimer’s disease -induced groups.

miRNA	HT-22		HT-22 + Aβ	
	Average	Standard deviation	Average	Standard deviation
miR-106b	856.707	109.67	56.07	25.924
miR-146b	35.24	15.226	535.924	23.458
miR-181a	15.26	13.731	315.226	34.147
miR-200a	23.31	13.712	233.731	45.631
miR-200c	34.12	13.501	234.712	51.672
miR-29	23.01	13.48	123.501	34.721
miR-124b	313.8	13.457	113.48	23.632
miR-34a	893.57	13.45	93.457	16.341
miR-200b	763.15	13.35	63.45	10.162
miR-133	151.75	23.277	53.35	9.127
miR-26	43.27	13.23	43.277	8.237
miR-128	133.23	23.118	33.23	6.813
miR-16	3.118	13.098	23.118	6.118
miR-153	23.098	3.064	363.980	4.115
miR-286	213.64	1.88	13.064	2.381
miR-7	612.8	2.831	12.88	3.314
miR-24	210.31	2.754	10.831	0.154
miR-134	71.54	2.691	11.754	0.071
miR-27	19.691	2.671	9.691	0.151
miR-176	37.671	2.669	7.671	0.034
miR-86	36.669	2.651	6.669	0.161
miR-87	64.651	2.595	4.651	0.027
miR-243	23.595	2.542	3.595	0.123
miR-144	2.542	2.499	2.542	0.265
miR-327	22.499	2.412	2.499	0.122
miR-289	11.412	2.382	1.412	0.012
miR-93	10.382	2.382	1.382	0.014
miR-386	4.382	2.366	0.382	0.076
miR-143	20.366	2.352	0.366	0.021
miR-145	4.352	2.301	0.352	0.011
miR-144	10.301	2.286	0.301	0.062
miR-155	568.286	2.247	0.286	0.027
miR-489	1.247	2.214	0.247	0.021

**Table 2:** Comparison of levels of inflammatory mediator antigens in plasma of control and Alzheimer’s disease animal’s model.

Antigen	Non-Tg mice		APP/PS/APoE KD	
	M	SD	M	SD
Aβ_1–42_ monomer	26.07	5.924	728.707	95.924
Aβ_1–42_ oligomer	5.24	1.226	635.924	35.226
UCHL1	35.26	5.731	425.226	53.731
NLRP3	33.31	13.712	653.731	63.712
Tau	2.12	0.501	734.712	32.501
STAT3	13.01	3.48	421.501	53.48
SORL1	15.8	3.457	323.48	21.457
Clusterin	21.57	3.45	393.457	23.45
ApoE3	323.15	42.35	263.45	43.35
ApoE4	300.75	31.277	364.35	11.277
Nogo-A	11.27	3.23	432.277	32.23
Visfatin	23.23	2.118	413.23	33.118
BNDF	243.118	23.098	76.118	6.098
GFAP	17.98	4.064	523.098	52.064
IL1-β	33.64	2.88	343.064	41.88
IL4	12.8	5.831	412.88	22.831
Caspase1	50.31	7.754	610.831	52.754
IL18	41.54	12.691	611.754	32.691
TNFα	39.691	2.671	419.691	22.671
p300	57.671	12.669	527.671	62.669
StandardA5M	842.669	92.651	896.669	32.651
Serum-free-media	14.651	5.595	12.651	3.595
Negative control	20.595	5.542	21.595	4.542
Albumin	761.542	22.499	690.542	32.499

Based on this approach, we selected miR-106b, miR-146b, miR-181a, miR-200a, miR-34a, miR-124b, miR-153, and miR-155 as markers for comparison with the control group and selected the Aβ_1–42_ monomer, Aβ_1–42_ oligomer, UCHL1, NLRP3, Tau, STAT3, SORL1, Clusterin, APOE3, APOE4, Nogo-A, IL-13, and Visfatin to serve as AD- and inflammation-related markers.

### Expression levels of AD-specific markers *in vitro*

**Figure 3:**
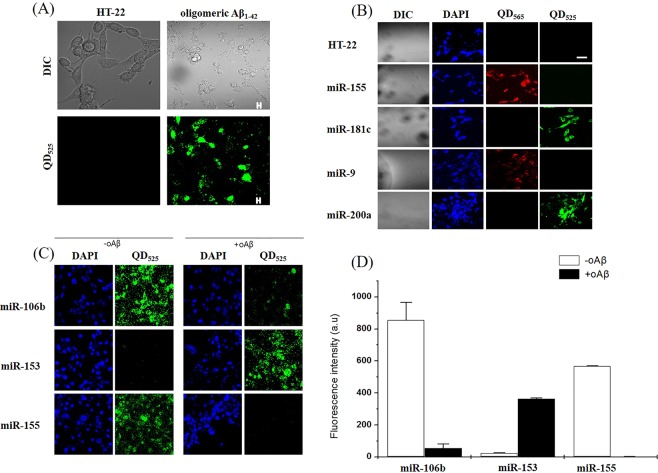
Expression and co-localisation of Aβ_1–42_ oligomer and QD_525_ (fluorescent nanoparticle) in HT22 cells. (**A**) Photograph of miRNA expression associated with inflammation when an AD-induced oligomeric amyloid peptide was treated in HT22 cells. A confocal electron microscope was used to measure the green and red fluorescence compared to that of the control. (**B**) The miR-155, miR-181c, miR-9 and miR-200a target nanocomposites were prepared. The oligomeric amyloid peptide was then added to the untreated control and to the treated group. The results are shown in photographs of the red and green fluorescence. (**C**) miR-106b, miR-153, and miR-155, which are already known to control AD, had large changes from the control group in the screened miRNA group. C also shows changes in the miRNA fluorescence when the HT22 cells were treated with and without oAβ, and the fluorescence intensity is expressed as a numerical value in a graph. (**A**–**C**) show that miRNA target fluorescent nanoparticles for AD diagnosis kits were successfully produced. The results were consistent with previous screening results.

Previously published studies on fluorescent nanoparticles have revealed the immunofluorescence reactivity in SH-SY5Y cells^24^. Therefore, we proceeded with HT22 cells and preincubated them in a 35-mm culture dish overnight. The HT22 cells were treated with 5 μM oligomer Aβ_1–42_ for 2 hours to observe specific target factors. To obtain *in vitro* fluorescence images of miR-155, miR-153a, miR-106b and miR-181c (mature type), confocal microscopy was performed during the incubation period of HT22 cells. The HT22 cells were fixed with a 4% formaldehyde solution containing 4′, 6′-diamidino-2-phenylindole dihydrochloride (DAPI) solution and then observed with a confocal microscope in a 35-mm culture dish. The normal HT22 cells are colourless, the DAPI-marked nuclei are blue, QD525 is indicated by red and green fluorescence, and the fluorescence contained in QD565 indicates a fluorescent nanoparticle. The oligomer Aβ_1–42_ exhibited green fluorescence and the fluorescence signals of miR-155, miR-181c, miR-9 and miR-200a (Fig. 3A,B). The mature miRNA, which is overexpressed in the cell, is bound to the miRNA molecular image sensor, and the quencher is dissociated from the molecular beacon, which is confirmed when the red or green fluorescence signal is increased. In particular, the green and red fluorescence signals were confirmed to be independently located at different positions within the cell so that the miRNA molecular image showed dissociation of the quencher.

miR-106b, miR-153, and miR-155, which are known to regulate AD, show a large difference in oAβ treatment in screening among miRNA groups^36–38^. The fluorescence of HT22 cells treated with and without oAβ was compared, and the same results as those in the screening were obtained (Fig. 3C).

### Expression levels of AD-specific markers *ex vivo*

**Figure 4:**
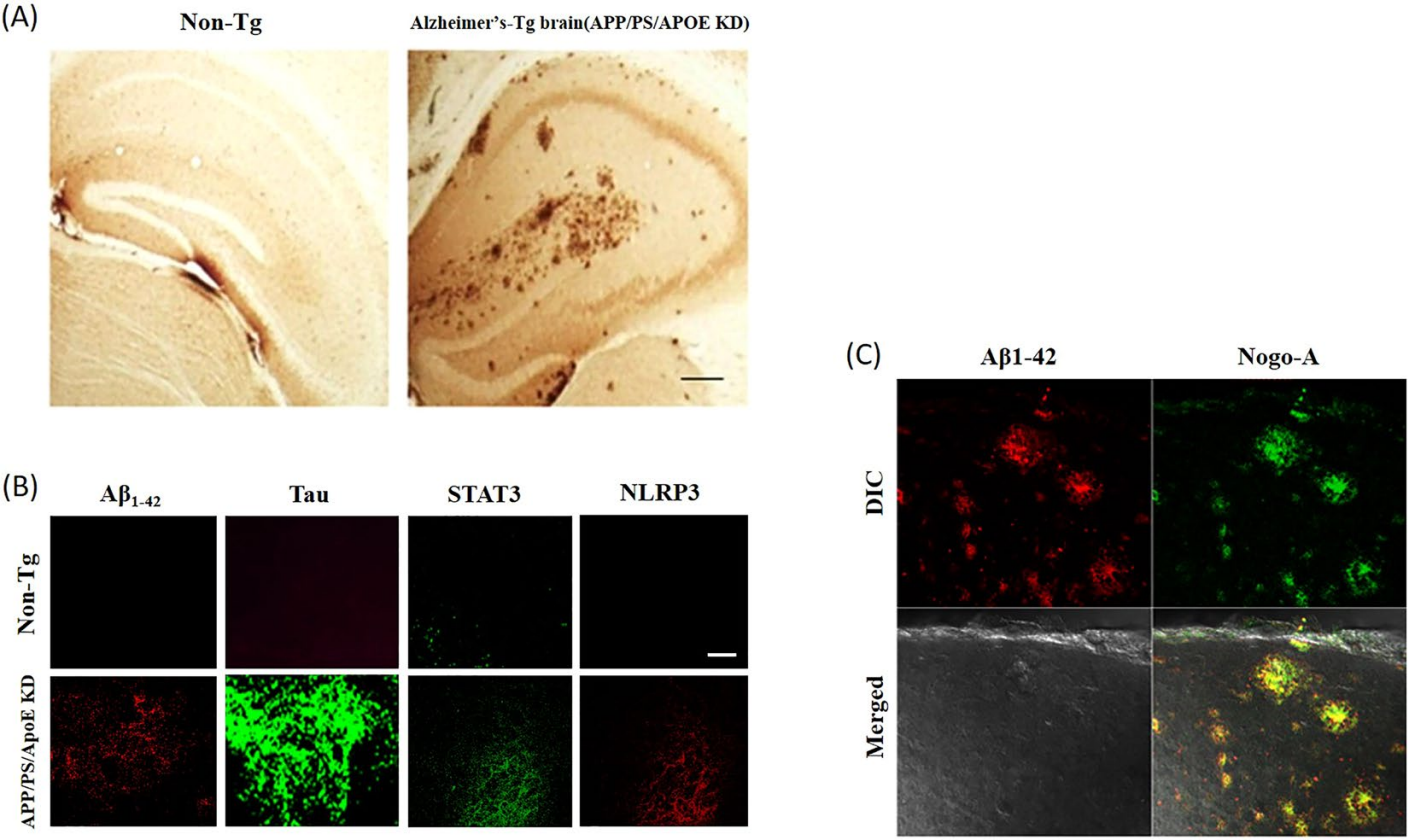
Expression of inflammation and AD biomarkers in APP/PS/ApoE KD Tg and non-Tg mice. (**A**) Micrograph showing the plaque formation of hippocampal tissues in normal mice (non-Tg) and AD-induced model mice (APP/PS/ApoE KD mouse). (**B**) Image of an immunocytochemical analysis of the expression levels of Aβ_1–42_, STAT3, Tau, and NLRP3 in Alzheimer’s disease progression in animal brain tissue. (**C**) Image of an immunocytochemical analysis showing the expression pattern of Aβ_1–42_ and Nogo-A expressed in the brain tissue of AD-induced model mice (APP/PS/ApoE KD mouse).

Recently, a study claimed that alteration of autophagy-targeting miR-124 led to downregulated BACE1 in APP/PS1 transgenic mice^39^. To diagnose inflammation and AD, a control (non-Tg mouse), an ApoE knockdown mouse (female, 24 weeks old) model for early inflammation, an APP/PS knockdown mouse group (40 weeks of age), and an APP/PS/ApoE KD mouse (Fig. 4A) (female, 12, 24 weeks old) model were used for severe AD. The cervical spines of the animals were removed, and part of the occipital lobe was excised. After removing the brain tissue, it was carefully washed with PBS (phosphate buffer), fixed with 4% PFA (para-formaldehyde) for 12 hours at 4 °C and cut at a thickness of approximately 10 μm with a vibratome. The samples were collected and placed in 24 wells. Then, the molecular image sensor nucleic acid complex prepared for the fixed tissue slice was dissolved in PBS and mixed with the anti-Aβ_1–42_, anti-Tau, anti-STAT3 and anti-NLRP3 antibodies. The fluorescent nanoparticles were added at 2 pmol each and reacted at 4 °C for 12 hours. A previous report demonstrated that Tau pathology and memory impairment are enhanced by showing miR-132/212 deficiency in 3xTg-AD mice^40^. As a result, no signals due to Aβ_1–42_, Tau, STAT3 and NLRP3 were observed in the control group, whereas red fluorescence due to Aβ_1–42_ and NLRP3 in the APP/PS/ApoE KD mouse was very strong, and green fluorescence by STAT3 and Tau was strong (Fig. 4B). On the other hand, to determine whether the signals were due to Aβ_1–42_ and Nogo-A and whether they were colocalised in the APP/PS/ApoE KD mouse, fluorescent nanoparticles containing QD565 that showed red fluorescence under a confocal microscope and the fluorescent Nogo-A nanoparticle containing QD525 that showed green fluorescence were detected (Fig. 4C). The results confirmed that Aβ_1–42_ and Nogo-A, which is a nerve paralytic protein, were expressed at the same location.

### Quantification of inflammation and AD-inducing target factors using molecular image sensors

**Figure 5:**
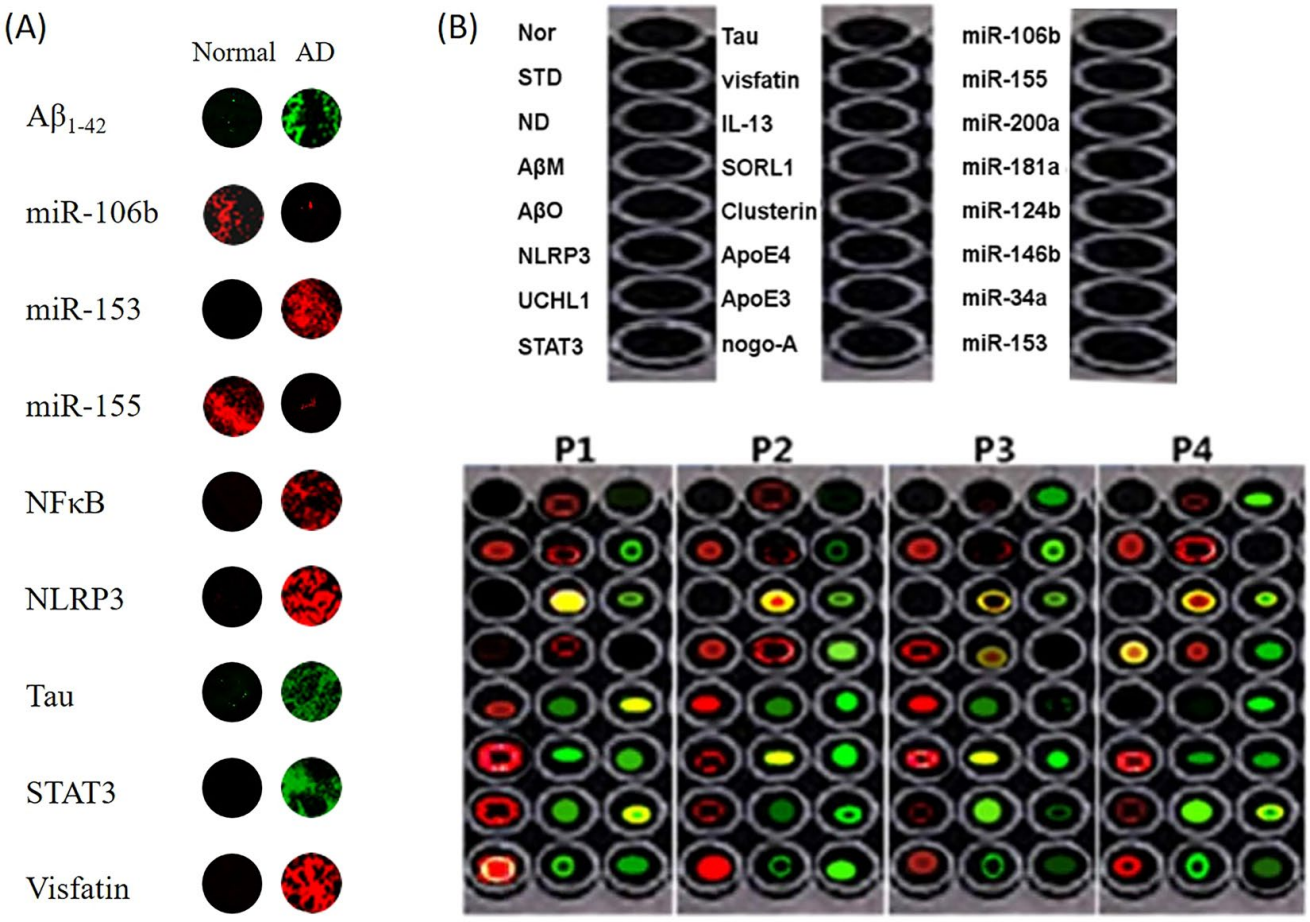
Human sample analysis using a molecular beacon NANP sensor. First, we want to confirm the applicability of *ex vivo* fluorescence in humans. (**A**) Profiling data for an AD-associated molecular beacon NANP sensor for normal and AD patients (ADAMBENA). Aβ_1–42_ miR-106b, miR-153, miR-155, NFκB, NLRP3, Tau, STAT3, and Visfatin were used as markers in two plasma samples from each normal and AD patient. (**B**) The profiling data for an AD-associated molecular beacon NANP sensor for 4 AD patients (ADAMBENA). miR-106b, miR-155, miR-200a, miR-181a, miR-124b, miR-146b, miR-34a, and miR-153 were used as markers in miRNA, and Aβ_1–42_ monomer, Aβ_1–42_ oligomer, NLRP3, UCHL1, STAT3, Tau, Visfatin, IL-13, SORL1, Clusterin, ApoE4, ApoE3, and Nogo-A were used as markers in four plasma samples from the AD patients.

First, we made nanoparticles to confirm the applicability of *ex vivo* fluorescence in humans. The Aβ_1–42_ oligomer miR-106b, miR-153, miR155, NFκB, Tau, STAT3, and Visfatin were used as markers in two plasma samples from each normal and AD patient. Four plasma samples were separated. 96-well container was blocked with BSA (0.1% BSA of sample was added to the each 96-well container). Then, 10 μl was dispensed into a 96-well container with a streptavidin-coated surface, and the molecular beacon prepared above was treated and then immediately observed or quantified with an LAS-4000 confocal microscope (Fig. 5A). As a result, the same results as those *in vitro* were obtained, confirming the applicability of fluorescence in humans.

miR-106b, miR-155, miR-200a, miR-181a, miR-124b, miR-146b, miR-34a, and miR-153 were used as markers for miRNA, and Aβ_1–42_ monomer, Aβ_1–42_ oligomer, NLRP3, UCHL1, STAT3, Tau, Visfatin, IL-13, SORL1, Clusterin, ApoE4, ApoE3, and Nogo-A were used as markers in four plasma samples from AD patients. The samples were obtained from the plasma of a female patient (P1~P4) in her seventies, and all four patients with known AD were selected by clinical diagnosis^41^. Four plasma samples were separated. 96-well container was blocked with BSA (0.1% BSA of sample was added to the each 96-well container). Then, 10 μl was dispensed into a 96-well container with a streptavidin-coated surface, and the molecular beacon prepared above was treated and then immediately observed or quantified with an LAS-4000 confocal microscope or fluorescence spectrophotometer (Fig. 5B and Table 3). In interpreting the results, the levels of Tau and Aβ_1–42_ oligomers increase with the severity of AD^42^. Thus, P1 has high levels of Tau and Aβ_1–42_ oligomers and likely has severe AD. In contrast, P2 and P3 are likely at the early stages of AD because of the low levels of Tau as well as the high levels of Aβ_1–42_ oligomers. P4 likely has MCI because the Tau levels are slightly high and the Aβ_1–42_ oligomer levels are very low.

**Table 3:** The level of Alzheimer’s disease -specific markers in plasma of patients with Alzheimer’s disease.

Patients	The marker and its plasma level(Unit arbitrary fluorescence intensity, X100)							
	No sample	average A	BSA	Aβ monomer	Aβ oligomer	NLRP1	UCHL1	STAT3
	Tau	Visfatin	IL-13	SORL1	Clusterin	ApoE4	ApoE3	Nogo-A
	miR-106b	miR-155	miR-200a	miR-181a	miR-124b	miR-146b	miR-34a	miR-153
P1	12.2 ± 0.1	255 ± 23	10.2 ± 0.5	0.2 ± 0.5	200 ± 15	412 ± 35	360 ± 12	650 ± 52
	180 ± 12	132 ± 12	890 ± 32	160 ± 21	150 ± 31	210 ± 5	190 ± 32	190 ± 5
	30 ± 5	200 ± 5	185 ± 11	23 ± 5	750 ± 25	300 ± 11	620 ± 42	190 ± 15
P2	ND	210 ± 2	0.2 ± 0.5	230 ± 11	200 ± 5	130 ± 25	120 ± 5	450 ± 12
	14 ± 5.22	120 ± 25	920 ± 16	190 ± 2	140 ± 20	650 ± 100	130 ± 5	150 ± 25
	14 ± 5	110 ± 3	145 ± 21	450 ± 23	370 ± 78	520 ± 100	200 ± 23	250 ± 45
P3	21 ± 5	220 ± 35	12 ± 3	200 ± 13	220 ± 14	190 ± 21	90 ± 4	100 ± 6
	12 ± 3	50 ± 6	310 ± 21	280 ± 16	190 ± 31	510 ± 3	420 ± 62	220 ± 41
	210 ± 35	250 ± 32	180 ± 43	14 ± 6	50 ± 2	290 ± 21	90 ± 2	40 ± 2
P4	21 ± 2	210 ± 2	16 ± 2	620 ± 90	21 ± 1	310 ± 21	110 ± 2	220 ± 13
	70 ± 1	230 ± 32	510 ± 23	190 ± 54	16 ± 5	150 ± 21	310 ± 23	230 ± 15
	190 ± 21	15 ± 4	230 ± 41	200 ± 11	220 ± 11	180 ± 31	310 ± 41	110 ± 3

In addition, P2 and P1 are expected to have more severe AD than P3 and P4 according to the results for miR-106b, which is involved in the regulation of AD and is reduced in oAβ-treated mice^36^. P2 and P1 are also expected to have more severe AD than P3 and P4 according to the results of miR-153, which is involved in the regulation of AD and is reduced in oAβ-treated mice^37^. As a result, P1 and P2 have severe AD, and P3 and P4 are expected to have early-stage AD or MCI. These results were the same as the clinical diagnoses.

## Discussion

MCI is considered to be the transition state between normal and AD, and determining the existence of brain amyloid deposits is the most important factor for determining the MCI stage for diagnosing AD. Disease-regulating therapy for AD has led to a need for biomarkers to identify prodromal AD and the early stages of AD. Therefore, we planned to make an AD diagnosis kit using fluorescent nanoparticles. As described above, the present data support a mechanism for the early diagnosis of AD. We selected miRNAs and inflammatory mediators to serve as molecular imaging sensors for detecting AD. Originally, miR-200a, miR-181a, miR-124b, miR-146b, miR-34a, miR-106b, miR-153 and miR-155, which are specifically expressed in astrocytes, glial cells, and hippocampal neurons, were selected for microRNAs. Next, amyloid-beta monomer, amyloid-beta oligomer, UCHL1, NLRP3, Tau, STAT3, SORL1, Clusterin, ApoE3, ApoE4, Nogo-A and Visfatin were selected as factors associated with inflammation and AD.

In other recent studies, miRNA levels in serum have been shown to be meaningful not only for the brain but also for several other pathologies^43–45^. We therefore selected miRNAs as well as existing Alzheimer’s disease-specific antigens. As earlier studies have demonstrated, loading nanoparticles and miRNA detection via fluorescent immunoreactivity were successful^17,28^. We demonstrated the detection ability of our fluorescent nanoparticles *ex vivo* as well as *in vitro*. For *ex vivo* analysis, we checked the binding ability of fluorescent nanoparticles and AD markers as well as inflammatory markers. The immunofluorescent reactivity revealed the expression levels, which showed no signals in normal mice, while the expression levels were significantly increased in APP/PS/ApoE KD transgenic mice. Furthermore, analysis of the immunoreactivity of fluorescent nanoparticles Q565 and Q525 revealed that mature miRNA, which is overexpressed in the cells, is bound to the miRNA molecular imaging sensor in HT22 cells.

We manufactured a molecular beacon-based sensor capable of detecting miRNA and 12 antigens, according to the method described above. We applied this approach to the plasma of four patients with different degrees of AD to confirm whether this technique can be used in a diagnostic kit for AD to determine the progress of AD through reactions with plasma obtained from an actual human. As a result, we confirmed that the abovementioned markers can be used for diagnosing the degree of AD in patients. According to the patient plasma results, miR-106b and miR-153 showed a significant expression pattern and could be used as early diagnostic markers to confirm the progress of AD. In addition, the levels of miR-124b, miR-34a, and miR-153 were significantly lower in the third patient than in the other three patients, which suggests that there was potential for molecular markers to confirm the degree of progression. However, among miR-106b, miR-153, and miR-155, which were previously known to modulate AD, miR-155 did not reveal a clear expression pattern in the results for the four patient plasma samples. Because this experiment included only four patients, we suggest that more experimental analyses with a larger sample population are needed to find a more accurate pattern of AD diagnosis. Therefore, we plan to use the AD diagnosis kit to analyse the results for more patients with AD next year.

Based on the above results, we concluded that the physiological response genetically reflects the early stages of inflammation *ex vivo* before the appearance of a phenotype that does not appear in the early stages of inflammation. If the miRNA and antigens related to AD are selected as biomarkers, the molecular image sensor discussed in this study can be used as part of a useful kit for the early diagnosis of AD. Therefore, the accuracy of the diagnosis can be further improved, and this improved early diagnostic kit will be helpful for the prevention and treatment of AD in the future.

## Materials and Methods

### Fluorescent nanoparticle preparation (miRNA)

A 5′-end oligonucleotide (TCGCTGT) capable of forming a stem at the 5′-end of the probe oligonucleotide, which is the complementary nucleotide of mature miR-155, miR-181a, miR-124b, miR-146b, miR-34a, miR-106b, miR-153, and miR-155, was used. A quantum dot (QD565) with red fluorescence was attached to the 5′-end of the 5′-end oligonucleotide. A customised nucleic acid molecular imaging sensor had an oligonucleotide linked to an arbitrary sequence (GTCGCTTT) that functioned as a switch at the 3′-end of the probe oligonucleotide (Bioneer, Korea). The method used a phosphite triester that connects phosphodiester bonds forming the backbone of DNA structures using -cyanoethyl phosphoramidite developed by Koster^46^. This method allows the desired oligonucleotide to be synthesised with high efficiency (synthesis efficiency >98%). In addition, the linker region has a nucleic acid sequence (CAGCG) that is capable of complementary binding to a nucleic acid molecule of a molecular image sensor and is biotin-bonded at the 5′-end. A nucleic acid switch molecule that is linked to BHQ2 (Black Hole Quencher 2) at the 3′-end of the linker region as a quencher was also customised using the phosphite triester method (Bioneer, Korea). Biotin acts as a stabiliser for the molecular imaging sensor nucleic acid molecule and the switch nucleic acid molecule, which forms a stem-loop type complex on the reaction container or microarray coated with streptavidin. The molecular imaging sensor nucleotides (MISNs) capable of detecting whole mature mouse (mma) and human (hsa) miRNA and the structure of a switch nucleic acid molecule capable of turning the MISN signal on and off are shown in Table 4.

**Table 4:** Structures of MISN and structure of switch nucleic acid molecules.

miRNA	Structure	Sequence number
mmu miR-34a	5′-QD_565_-TCG CTGT ACAACCAGCTAAGACACTGCCA GTCGCTTT-3′	1
mmu mir-106b	5′-QD_565_-TCG CTGT ATTTCACGACTGTCACGTCTA GTCGC TTT-3′	2
mmu miR-124b	5′-QD_565_-TCG CTGT GGCATTCACCGCGTGCCTTA GTCGCTTT-3′	3
mmu miR-146b	5′-QD_565_-TCG CTGT AGCCTATGGAATTCAGTTCTCA GTCGCTTT-3′	4
mmu mir-153	5′-QD_565_-TCG CTGT AACGTATCAGTGTTTTCACTAG GTCGC TTT-3′	5
mmu mir-155	5′-QD_565_-TCG CTGT AATTACGATTAACACTATCCCCA GTCGC TTT-3′	6
mmu miR-181a	5′-QD_565_-TCG CTGT ACTCACCGACAGCGTTGAATGTT GTCGCTTT-3′	7
mmu miR-200a	5′-QD_565_-TCGCTGT ACATCGTTACCAGACAGTGTTA GTCGCTTT-3′	8
hsa miR-34a	5′-QD_565_-TCGCTGT ACAACCAGCTAAGACACTGCCA GTCGCTTT-3′	9
hsa mir-106b	5′-QD_565_-TCGCTGT ATTTCACGACTGTCACGTCTA GTCGC TTT-3′	10
has miR-124b	5′-QD_565_-TCGCTGT GGCATTCACCGCGTGCCTTA GTCGCTTT-3′	11
hsa miR-146b	5′-QD_565_-TCGCTGT AGCCTATGGAATTCAGTTCTCA GTCGCTTT-3′	12
has mir-153	5′-QD_565_-TCGCTGT AACGTATCAGTGTTTTCACTAG GTCGC TTT-3′	13
hsa mir-155	5′-QD_565_-TCGCTGT AATTACGATTAGCACTATCCCCA GTCGC TTT-3′	14
hsa miR-181a	5′-QD_565_-TCGCTGT ACTCACCGACAGCGTTGAATGTT GTCGCTTT-3′	15
hsa miR-200a	5′-QD_565_-TCGCTGT ACATCGTTACCAGACAGTGTTA GTCGCTTT-3′	16
Switch nucleic acid molecule	5′-biotin-AGCGA-BHQ2–3′	

(The underlines mean target sequence).

For the reaction, 500 pmol of the switch nucleic acid molecule containing a quencher (BHQ2) and 500 pmol of MISN containing QD565 were mixed in 1 ml of hybridisation buffer (1x TE buffer, 100 mM NaCl, pH 7.8) and incubated at a temperature of 93 °C for 5 minutes. After cooling slowly to a temperature of 4 °C, the molecular sensing nanoparticles were synthesised.

### Fluorescent nanoparticle preparation (antibody)

The structure of the antibody-binding type molecular sensing nanoparticles formed a molecular imaging sensor nucleotide-antibody complex (MISNAC) and a nucleic acid switch molecule as follows (the sequence number indicates the entire nucleic acid sequence of the right fragment based on the antibody),

MISNAC:

5′-QD565-TCGCTG-CONH-antibody-CONH-TATTTCACGACTGTCACGTCTAGTGCTTT-3′

(sequence number 17, the underlined text means the target sequence).

nucleic acid switch molecule:

5′-biotin-CG-BHQ2–3′

A left arm of MISN containing 2 μg antibody and QD565 was custom made, and a right arm of MISNAC containing a random sequence (GTGCTTT) for the 3′-end of the oligonucleotide performing a switch function was also custom made using the phosphite triester method (Bioneer, Korea). Carboxylic group (COOH) of antibody in left arm reacts with 1-ethyl-3-(3-dimethylaminopropyl)carbodiimide hydrochloride (EDC) and convert to the o-Acylisourea Active Ester. Then add primary amine of right arm to conjugated an amide bond between right arm and left arm. After reacting for 1 hour under the specified conditions (EDC, Tris-HCl, pH < pI of antibody R group, 0.1 M EDTA, 5:1 oligonucleotide-to-antibody ratio, RT), the samples were centrifuged at 15,000 rpm for 10 minutes, and the supernatant was discarded^47^. Next, the pellets were collected and dissolved in a phosphate buffer solution to prepare MISNAC. Then, 500 pmol of the switch nucleic acid molecule containing a quencher (BHQ2) and 500 pmol of MISNAC containing QD565 were mixed in 1 ml of hybridisation buffer (1x TE buffer, 100 mM NaCl, pH 7.8) and incubated at a temperature of 93 °C for 5 minutes. After cooling slowly to a temperature of 4 °C, the antibody-bound molecular sensing nanoparticles were synthesised.

### Animals

To perform the inflammation and AD study, the control group (non-Tg mice) ApoE KD mice (female, 24 weeks old) and the APP/PS mouse group (females that served as a model of mild cognitive disability from initial inflammation, 40 weeks old) were anaesthetised as previously described with a few changes^48,49^. And then fixed with a transcardiac perfusion of 4% paraformaldehyde (PFA). After disconnecting a cervical anatomy port by cutting a portion of the back of the head, a portion of the skull and the brain were removed and washed carefully with PBS (phosphate-buffered saline). The brain tissue was fixed at 4 °C for 12 hours in 4% PFA (para-formaldehyde) and cut to a 10-μm thickness with a vibratome to obtain the hippocampal and cortical portions, which were collected in a 24-well plate.

### Antibodies

Anti-APP, anti-Aβ_1–42_ monomer, anti-Aβ_1–42_ oligomer, anti-Tau, anti-STAT3, anti-NLRP3, anti-ApoE4, anti-Nogo-A, anti-IL-13, anti-TNFα, anti-GFAP, anti-SORL1, anti- UCHL1, anti-Visfatin and anti-Clusterin were used (Santa Cruz, USA).

### Cell culture

The HT22 mouse hippocampal cell line (ATCC (American Type Culture Collection)) was used to observe the target-specific factor for hippocampal neurons, and cells were cultured from the hippocampal neurons. Then, the HT22 cells were incubated with 5 μM oligomer Aβ_1–42_ for 2 hours, and the ATCC cells were cultured. To obtain the expression of miR-155 (mature type), miR-153a (mature type), miR-106b (mature type), miR-181c, and miR-9 *in vitro*, fluorescent images of miR-200a were captured for the HT22 cells during the culture period using a confocal microscope.

### Immunofluorescence staining

Immunofluorescence staining was performed to distinguish the expression levels of the various biomarkers we measured earlier^50,51^. In brief, the slides were dried before staining at room temperature and then washed with PBS (0.01 mM for 10 minutes). The slides were incubated with a primary antibody for a whole night at 4 °C. The slides were then incubated with a secondary antibody (rabbit/mouse) followed by DAPI treatment and covered with glass coverslips using mounting medium. Confocal laser microscopy (FluoView FV 1000 MPE) was used to collect the images.

### AD clinical assessment

PET (positron emission tomography) images were analysed, and all subjects were categorised by neurologists at Bundang Seoul National University Hospital using the CERAD-K program of AD Clinical Assessment of Korean Association for Dementia to identify the normal persons and AD patients^52,53^.

### Compliance with ethical standards

Total of six human serum plasma samples including one healthy and five patients were obtained. All human subjects were female in age ranges from 70–75 years informed consent was obtained from all participant. All experimental protocols were approved Bundang Seoul National University Hospital IRB: B-1403/242-301 (Institutional Review Board). All experiments were performed in accordance with Bundang Seoul National University Hospital IRB guidelines. All animal studies were approved by Institutional Animal Care and Use Committee in Bundang Hospital in Seoul National University (IACUC Protocol, BA1508-183-054-01), South Korea. Efforts were made to minimize the number of mice used and to reduce their suffering. The experimental approaches with mice were carried out according to the approved guidelines (Approval ID:125), and all protocols were approved by the IACUC of the Division of Life Science and Applied Life Science at GNU, South Korea (Approval number: GNU-181107-M0057).
